# Neonatal conjunctivitis at a Brazilian tertiary center: the current relevance of *Chlamydia trachomatis*

**DOI:** 10.5935/0004-2749.2023-0290

**Published:** 2024-10-31

**Authors:** Juliana Mika Kato, Eduardo Ferracioli Oda, Thaisa Silveira Barbosa, Flavia Rossi, Andre Mario Doi, Carolina dos Santos Lazari, Tatiana Tanaka, Joyce Hisae Yamamoto

**Affiliations:** 1 Departamento de Oftalmologia, Hospital das Clínicas, Faculdade de Medicina, Universidade de São Paulo, São Paulo, SP, Brazil; 2 Divisão de Laboratório Central, Hospital das Clínicas, Faculdade de Medicina, Universidade de São Paulo, São Paulo, SP, Brazil

**Keywords:** Conjunctivitis, Infant, newborn, diseases, Ophthalmia neonatorum, Chlamydia infections, Sexually transmitted diseases

## Abstract

**Purpose:**

The microbiology pattern of neonatal conjunctivitis has changed over time, and the incidence of gonococcal conjunctivitis is almost nil. This study aimed to determine the etiology of neonatal conjunctivitis cases referred to a tertiary health center in Brazil.

**Methods:**

From 2017 to 2020, conjunctival swabs were taken from neonates with clinical signs of conjunctivitis and tested with bacterial culture and polymerase chain reaction for *Neisseria gonorrhoeae* and *Chlamydia trachomatis.*

**Results:**

A total of 51 neonates were included in the 3-year study. Chlamydial conjunctivitis was diagnosed in 39 (76.5%) patients, and microbial growth was detected in 13 (25.5%) patients. The most isolated bacterium was *Staphylococcus epidermidis* (n=6, 11.8%), followed by other coagulase-negative *Staphylococcus* species (n=4, 7.8%) and *S. aureus* (n=2, 3.9%). One *S. aureus* isolate was resistant to oxacillin. There were no cases of gonococcal conjunctivitis. Ten (19.6%) patients showed polymerase chain reaction-negative *C. trachomatis* and negative bacterial culture test results.

**Conclusion:**

Findings show that *C. trachomatis* is the most common pathogen causing neonatal conjunctivitis. The high prevalence of *C. trachomatis* infection highlights the importance of screening and treating pregnant woman.

## INTRODUCTION

Neonatal conjunctivitis is defined as conjunctivitis occurring anytime during the first 30 days of life and is considered the most common infection during the neonatal period. Its incidence varies depending on the patient’s sociodemographic conditions. Neonatal conjunctivitis has traditionally been associated with *Neisseria gonorrhoeae* and *Chlamydia trachomatis* infection. However, Credè’s method of prophylaxis with a single drop of 2% silver nitrate solution immediately after birth has decreased the incidence of gonococcal conjunctivitis to almost nil^(1)^. Recent studies have identified *Staphylococcus aureus* and *S. epidermidis* as the most common bacterial species causing neonatal conjunctivitis^(2)^. Therefore, this study aimed to determine the etiology of neonatal conjunctivitis cases referred to a tertiary health center in São Paulo, Brazil.

## METHODS

From 2017 to 2020, conjunctival swabs were taken from neonates with clinical signs of conjunctivitis referred to the *Hospital das Clínicas, Faculdade de Medicina da Universidade de São Paulo* (HC-FMUSP). Symptoms included ocular discharge, hyperemia, and swelling of the eyelids. We tested the samples for the presence of *N. gonorrhoeae* using bacterioscopy with Gram staining, chocolate agar, and Thayer-Martin agar cultures and for the presence of *C. trachomatis* with polymerase chain reaction (PCR) using the Abbott Real Time CT/NG Amplification Kit (Abbott Molecular Inc., IL, USA)^(3)^. Epidemiological and clinical data were collected from the patients’ medical records. The recommended treatment was intramuscular injection of ceftriaxone (a single dose of 50 mg/kg) and azithromycin oral syrup (20 mg/kg/day) once daily for 3 days^(3)^. All cases were referred to the Pediatric Emergency Center of the HC-FMUSP for systemic evaluation.

This study was approved by the Institutional Ethics Committee of the HC-FMUSP.

## RESULTS

A total of 64 neonates were admitted to the Ophthalmology Emergency Center of the HC-FMUSP during the 3-year study period. Of these, 13 (20.3%) patients were excluded due to insufficient microbiological data. The main findings of the remaining 51 (79.7%) patients are presented in table 1. The patients’ mean age at presentation was 17.6 (range 7-33) days, and the average duration of symptoms was 8.9 (range 1-24) days. Most cases were vaginal deliveries (n=39, 76.5%), and the majority of the mothers (n=30, 58.8%) reported some complications during pregnancy, including urinary tract infections, premature rupture of membranes, or vaginal discharge.

**Table 1:** Main findings of neonates (n=51) presenting with conjunctivitis at the HC-FMUSP^a^ from 2017 to 2020

Description	Value
Age (days) at presentation, mean (range)	17.6 (7-33)
Symptom duration, mean (range)	8.9 (1-24)
Type of delivery, n (%)	
Vaginal delivery	39 (76.5)
Cesarean section	5 (9.8)
Not informed	7 (13.7)
Birth weight (g), mean (range)	3185 (2210-5070)
Low birth weight (<2500), n (%)	4 (7.8)
Normal (2500-4000), n (%)	27 (53.0)
Macrossomia (>4000), n (%)	1 (2.0)
Not informed, n (%)	19 (37.2)
Gestational age, n (%)	
Preterm	4 (7.8)
Term	34 (66.7)
Not informed	13 (25.5)
Pregnancy complications, n (%)	
Yes	30 (58.8)
No	10 (19.6)
Not informed	11 (21.6)
Silver nitrate eye drops as prophylaxis, n (%)	
Yes	17 (36.2)
No	5 (10.6)
Not informed	25 (53.2)
Positive PCR^b^ for Chlamydia trachomatis, n (%)	39 (76.5)
Culture result, n (%)	
Staphylococcus epidermidis	6 (11.8)
Coagulase-negative Staphylococcus spp.	4 (7.8)
Staphylococcus aureus	2 (3.9)
Corynebacterium sp.	1 (2.0)
Viridans group Streptococcus	1 (2.0)
Citrobacter freundii complex	1 (2.0)
Eikenella corrodens	1 (2.0)
Negative	38 (74.5)

a HC-FMUSP: Hospital das Clínicas, Faculdade de Medicina da Universidade de São Paulo.

b PCR: polymerase chain reaction.

Chlamydial conjunctivitis was diagnosed with PCR in 39 (76.5%) patients, and microbial growth was detected in 13 (25.5%) patients. The most isolated bacterium was *S. epidermidis* (n=6, 11.8%), followed by other coagulase-negative *Staphylococcus* species (n=4, 7.8%) and *S. aureus* (n=2, 3.9%). One *S. aureus* isolate was resistant to oxacillin (methicillin-resistant *Staphylococcus aureus* [MRSA]). There were no cases of gonococcal conjunctivitis. In addition, 12 (23.5%) patients showed PCR-positive *C. trachomatis* and also positive bacterial culture results, including 3 (25.0%) multibacterial cases, while 10 (19.2%) patients showed PCR-negative *C. trachomatis* and negative bacterial culture results.

## DISCUSSION

In this study, *C. trachomatis* was the most common pathogen found to cause neonatal conjunctivitis, accounting for 76.5% of the cases. This prevalence markedly surpasses the reported range of 0%-46% in the literature^(4^,^5)^. Studies investigating *C. trachomatis* infection among women in Brazil have reported a prevalence of 12%-31%^(6)^. The HC-FMUSP, however, is a tertiary referral center and receives patients with high-risk pregnancies and a greater risk of complications compared to most other institutes.

Since *C. trachomatis* infection is associated with preg-nancy complications, such as premature rupture of membranes, preterm delivery, growth restrictions, and fetal loss, the estimated prevalence is understandable but alarming. Previous studies have used widely different methods of identifying *C. trachomatis* in neonatal conjunctivitis cases or did not even test for it. Detecting the etiological agent may have been difficult due to the unavailability of current technologies, such as PCR.

Studies on the microbiology of neonatal conjunctivitis have reported varying rates of culture positivity, ranging from 0% to 83%, as well as the prevalence of other coagulase-negative *Staphylococcus* species and *S. aureus*^(4^,^5)^. In this study, microbial growth was detected in 25.0% of the patients. These microorganisms, however, may be part of the normal ocular flora and might not be directly related to neonatal conjunctivitis. Distinguishing between a colonizing agent and a true pathogen is a challenge from a microbiological perspective, especially given the difficulty in sterile specimen collection from an unwilling newborn. The high rate of positive bacterial culture results in this study may also be related to the higher frequency of vaginal deliveries compared to cesarian deliveries.

Of the 51 cases, 19.6% tested negative for *C. trachomatis* and other bacteria. These cases could be due to viral pathogens, chemical irritants, or nasolacrimal duct obstruction. However, viral PCR is not routinely performed at the HC-FMUSP.

As neonates with untreated mothers have a 50% chance of developing conjunctivitis and up to a 20% chance of developing pneumonia, screening of pregnant women is strongly recommended. Silver nitrate solution does not prevent chlamydial conjunctivitis, and the evidence of antibiotic ointments decreasing the incidence of chlamydial conjunctivitis is sparse^(7)^.

The findings of this single-center study conducted in S**ã**o Paulo, Brazil, may offer insights into chlamydial conjunctivitis infection among the broader Brazilian population. We strongly advocate for routine screening and treatment of pregnant women during prenatal care in order to prevent both pregnancy-related and neonatal complications.
